# Colonization History, Host Distribution, Anthropogenic Influence and Landscape Features Shape Populations of White Pine Blister Rust, an Invasive Alien Tree Pathogen

**DOI:** 10.1371/journal.pone.0127916

**Published:** 2015-05-26

**Authors:** Simren Brar, Clement K. M. Tsui, Braham Dhillon, Marie-Josée Bergeron, David L. Joly, P. J. Zambino, Yousry A. El-Kassaby, Richard C. Hamelin

**Affiliations:** 1 Department of Forest and Conservation Sciences, The University of British Columbia, Vancouver, British Columbia, Canada; 2 Natural Resources Canada, Laurentian Forestry Centre, Quebec City, Quebec, Canada; 3 Département de Biologie, Université de Moncton, Moncton, New Brunswick, Canada; 4 USDA Forest Service, Coeur d'Alene Field Office, Coeur d'Alene, Idaho, United States of America; University of Nebraska-Lincoln, UNITED STATES

## Abstract

White pine blister rust is caused by the fungal pathogen *Cronartium ribicola* J.C. Fisch (Basidiomycota, Pucciniales). This invasive alien pathogen was introduced into North America at the beginning of the 20th century on pine seedlings imported from Europe and has caused serious economic and ecological impacts. In this study, we applied a population and landscape genetics approach to understand the patterns of introduction and colonization as well as population structure and migration of *C*. *ribicola*. We characterized 1,292 samples of *C*. *ribicola* from 66 geographic locations in North America using single nucleotide polymorphisms (SNPs) and evaluated the effect of landscape features, host distribution, and colonization history on the structure of these pathogen populations. We identified eastern and western genetic populations in North America that are strongly differentiated. Genetic diversity is two to five times higher in eastern populations than in western ones, which can be explained by the repeated accidental introductions of the pathogen into northeastern North America compared with a single documented introduction into western North America. These distinct genetic populations are maintained by a barrier to gene flow that corresponds to a region where host connectivity is interrupted. Furthermore, additional cryptic spatial differentiation was identified in western populations. This differentiation corresponds to landscape features, such as mountain ranges, and also to host connectivity. We also detected genetic differentiation between the pathogen populations in natural stands and plantations, an indication that anthropogenic movement of this pathogen still takes place. These results highlight the importance of monitoring this invasive alien tree pathogen to prevent admixture of eastern and western populations where different pathogen races occur.

## Introduction

Invasive fungal pathogens of trees represent a global threat to natural forests and tree plantations, resulting in economic losses and, in some cases, changes to ecosystem functions and a reduction in biodiversity [1–3]. Globalization and the increase in international trade, travel, and transport are largely responsible for this trend [4]. The fungal pathogen *Cronartium ribicola* J.C. Fisch (Basidiomycota, Pucciniales) causes white pine blister rust, a disease that affects all white pines (*Pinus* subsection *Strobus*). This invasive alien pathogen was introduced into North America and discovered at the beginning of the 20th century on pine seedlings imported from Europe and planted for reforestation in the USA and Canada. White pine blister rust is responsible for one of the worst forest disease epidemics recorded in the Northern Hemisphere. It can cause mortality rates approaching 100% in high hazard zones and has severely reduced survival, natural regeneration and reforestation efforts with white pines in North America [5–7].

The history of introduction of this pathogen is relatively well documented. Following extensive logging in the 19th century, there were intensive efforts made to replant the highly valuable eastern white pine (*Pinus strobus* L.). North American nurseries lacked the capacity to supply the number of seedlings required and millions of white pine seedlings were imported from Europe to North America for reforestation [8]. The pathogen, which had been known in Europe since the late 19th century, was accidentally introduced into North America on infected seedlings [5,8–14]. The introduction and colonization history of this pathogen in North America is expected to have left population genetic signatures that are influenced by the size and number of these introduction events.

Another important factor in shaping *C*. *ribicola* populations is the distribution of its hosts. This pathogen is a biotrophic heteroecious rust fungus; therefore, the presence of a live host and alternation between two unrelated hosts are required for the completion of its life cycle [11]. White pine blister rust can infect all North American white pine species and distribution of the pathogen tends to track the distribution of its hosts. White pines cover a wide latitudinal, longitudinal and elevational range, and at least one species of white pine is found in most coniferous forests [12–14]. In western North America there are six white pine species affected by *C*. *ribicola* that are distributed across a wide latitudinal and elevational range. Some of these species, such as *Pinus strobiformis* Engelm., occur in isolated patches, a factor that could impact pathogen dispersal and colonization. In addition, there is a broader range of alternate hosts, angiosperms in the families Grossulariaceae and Orobanchaceae, in western than in eastern North America [15–18].

The pathogen is also confronted with more extreme landscape and climatic variations in western than in eastern North America. *Cronartium ribicola* is found from New Mexico to Smithers in BC, spanning more than 22° of latitudinal range. The climatic conditions across that range vary from the northern limit of white pines at 55° latitude, with a mean temperature of 3.75°C and 180 mm of monthly snow fall, to high elevation pine sites in New Mexico, with a mean temperature of 24°C and 25 mm of monthly rain. These differences could result in varied selection pressure favoring local adaptation. This could combine with population disjunction and barriers to gene flow and contribute to shaping these fungal populations.

Understanding the patterns of introduction, colonization, population structure and migration of invasive alien pathogens is important [19]. Genetic analyses might inform forest managers about epidemiological patterns and dispersal ability and reveal incipient novel adaptations. In addition, molecular markers can be developed to track pathogen spread and identify sources so that further spread can be prevented [20]. The integration of genetic data coupled with geographic coordinates, host distribution and landscape features can provide a powerful dataset that yields insights into epidemiology and dissemination patterns and can help predict and prevent future spread [21]. Studying the patterns of genetic diversity at the landscape level could reveal local adaptations related to host distribution or landscape features as well as adaptation along latitudinal or altitudinal gradients, and could help detect and maintain or enforce barriers to migration. The objectives of this study were to characterize *C*. *ribicola* populations in North America using single nucleotide polymorphisms (SNPs) and evaluate the effect of landscape features, host distributions, climate and colonization history on the structure and variability of these pathogen populations.

## Methods

### Sampling of *Cronartium ribicola*

Most of the samples were collected from Canadian Crown land, for which no collection permits were required. In the cases where samples were obtained from private plantations, permissions were obtained from the owners. No sampling of endangered species was conducted. Samples of *C*. *ribicola* were collected between 1996 and 2010 across North America (S1 Table). A continent-wide sampling [22,23] was conducted at 76 sites, including natural stands and plantations of white pines. Sampling in British Columbia and Alberta was designed to explore landscape and host patterns in western North America. The sampling was structured to represent and pair, whenever possible, high and low elevation sites from coastal and interior regions of British Columbia and Alberta.

Sample collection was conducted as described previously [22]. Briefly, aeciospores were collected from mid-April to mid-June, depending on the location, prior to the opening of aecial blisters to ensure that there was no cross-contamination. This method allows direct sampling of unique dikaryotic individuals without the need for additional manipulations, an important feature since this fungus is a biotroph [22]. A toothpick was used to rupture individual aecial blisters and the aeciospores were collected in 1.5 mL microcentrifuge tubes. Whenever possible, at least three aecia per canker and ten trees per site were sampled. The samples were dried in desiccation chambers with a layer of water saturated with calcium chloride at the bottom of the chambers and then placed at -20°C for storage.

### DNA extraction and SNP genotyping

Total genomic DNA was extracted using the DNeasy Plant Mini kit (Qiagen Inc., Toronto, ON, Canada), with some modifications made to the manufacturer’s recommendations. The frozen spores were placed into microcentrifuge tubes along with a sterile Tungsten carbide bead, 500 μL of AP1 buffer, 1 μL of Reagent DX, and 1μL RNase A (100 mg/mL). Spores were mechanically disrupted using a Retsch Mixer Mill MM 400 (Qiagen, Alameda, CA, USA) for 2 min, placed in an 80°C water bath for 10 min followed by another 2 min in the Mixer Mill, and then boiled for 5 min to complete the disruption step. Once removed from the boiling water, 150 μL of AP2 buffer was added to the spore mixture, the tube centrifuged, and placed at -20°C for 10 min, and then centrifuged again for 5 min. The rest of the extraction follows the protocol provided in the Qiagen kit.

SNPs were first identified from an EST library and a draft genome of *C*. *ribicola*, and were then screened on a subset of 16 samples from eastern and western North American populations. A total of 31 polymorphic SNPs were identified that met the requirements for genotyping using an assay based on the iPLEX primer extension protocol on a MassARRAY Compact system (Sequenom, McGill University and Génome Québec Innovation Centre platform, Montréal, QC, Canada; http://genomequebec.mcgill.ca/centre.php) (S2 Table).

### Data analysis

Genetic analyses, including frequency- and likelihood-based methods, were performed to infer the population structure and determine the effects of landscape and other factors on *C*. *ribicola*. Tests for deviation from Hardy-Weinberg equilibrium were performed to determine if the data met assumptions of some of the analyses. A total of 66 populations, representing 1292 individuals, had population size n>4 and were used in frequency-based analyses. However, all 1336 individuals from 76 populations were included in the likelihood-based assignment analyses. Linkage disequilibrium (LD) was verified for non-random association of alleles at all loci using *Arlequin* [24]. For further analyses that are sensitive to LD, we reduced the dataset to 27 SNPs.

*GenAlEx* version 6.4 [25] was used to calculate allele frequencies, to test for deviation from Hardy-Weinberg equilibrium, and to perform a Principal Component Analysis (PCA). A pairwise Nei’s unbiased genetic distance matrix was generated in *GenAlEx*, and a neighbor joining tree analysis was performed with Ape version 3.1.1, an R package (http://ape-package.ird.fr/). Deficiency and excess of heterozygotes were estimated by an exact test implemented in Genepop version 4.0.10 [26].

The software *Structure* version 2.3.2 was used to infer population structure and to assign individuals to K clusters, based on genotypic data [27]. The analysis was conducted with the number of clusters set from K = 1 to 15, and each K was replicated 10 times, with a burn-in length of 100,000 generations followed by 800,000 generations to verify the likelihood values of each K value. Admixture (mixed ancestry) and independent allele frequency models were tested. The results did not differ; therefore, only results from the independent allele frequency model are presented. *Structure Harvester* (http://taylor0.biology.ucla.edu/struct_harvest/) was used to estimate the K value with the highest likelihood [28].

A spatial Principal Component Analysis (sPCA) was conducted using *Adegenet* version 1–3.6, an R package, to incorporate spatial coordinates within the PCA [29]. This approach has been proposed to identify cryptic spatial patterns that can be hidden when the genetic signal is weak. This analysis uses spatial information modeled explicitly through a connection network and incorporates Moran’s index of spatial autocorrelation [30]. We used Delaunay triangulation, which provided extended pairwise connectivity, as a connection network. The first positive eigenvalue component of the sPCA, corresponding to global structure, was plotted on a geographic map to examine the impact of landscape features or host distribution on the spatial genetic structure.

*Adegenet* was also used to test for isolation-by-distance. Correlation between genetic and geographic distance matrices was evaluated using a Mantel test. Because of the large genetic differentiation between the eastern and western populations, analyses of isolation-by-distance were also conducted separately within each cluster.

AMOVA, implemented in *Arlequin*, was used to determine the proportion of genetic variance attributed to different factors. AMOVA was used to investigate the proportion of variation attributed to regions (the eastern and western North American), type of sampling (plantations vs natural stands), landscape features (elevation, mountain ranges and proximity to the coast) and host pine species.

Identification of barriers to gene flow was conducted with the Monmonier algorithm implemented in *Adegenet*. We used Delaunay triangulation as connection networks and pairwise Euclidean distances to detect genetic boundaries among our geo-referenced populations. The analysis is designed to find the path through the strongest genetic distances between neighbors.

## Results

### Colonization history and host distribution influence white pine blister rust populations

**Fig 1:**
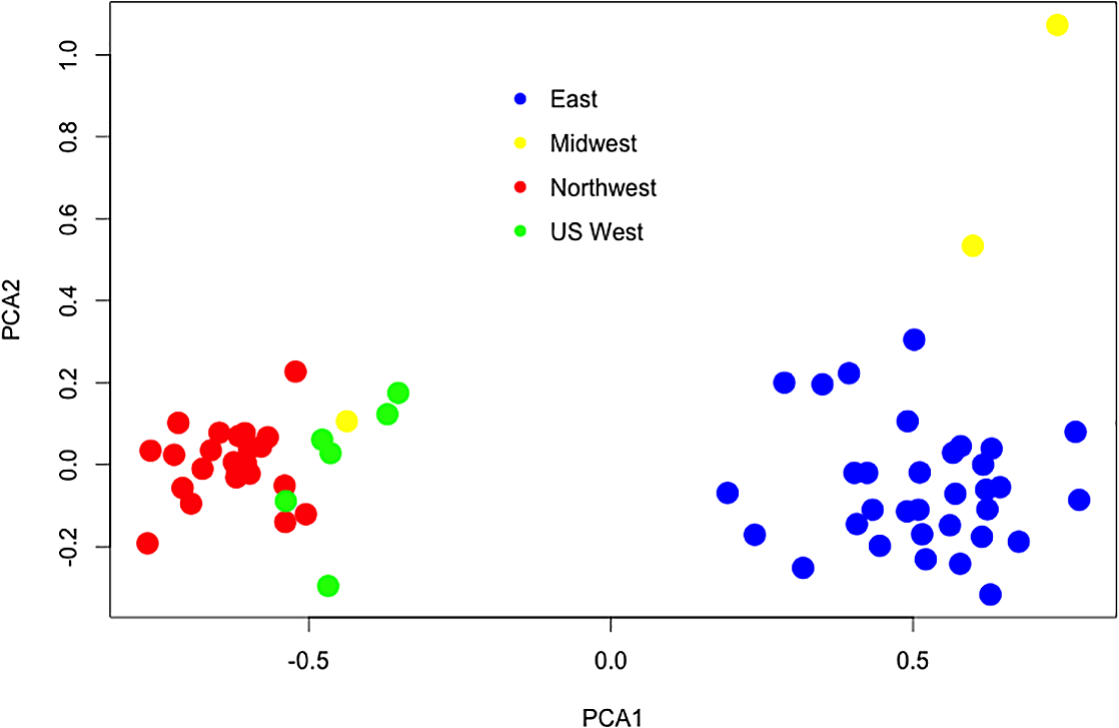
Principal component analysis using a pairwise population matrix of mean population codominant genotypic genetic distances among 66 North American populations of *Cronartium ribicola*. First and second components, accounting for 70% and 9% of the total genetic variability. B) First and third components, accounting for 70% and 7% of the total variability. East, Midwest, Northwest and US West refer to regions in S1 Table.

**Fig 2:**
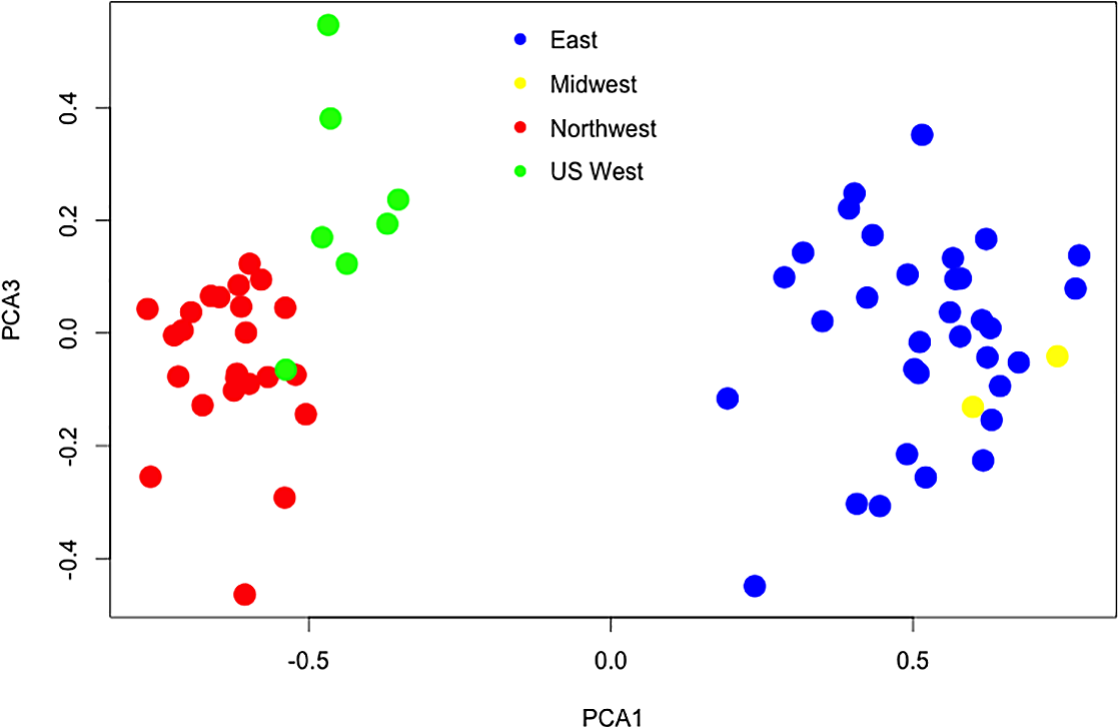
Principal component analysis using a pairwise population matrix of mean population codominant genotypic genetic distances among 66 North American populations of *Cronartium ribicola*. First and third components, accounting for 70% and 7% of the total variability. East, Midwest, Northwest and US West refer to regions in S1 Table.

The structure of North American *C*. *ribicola* populations is strongly influenced by geographic origin, with longitudinal and latitudinal patterns. The first axis of the PCA separates the populations into eastern and western clusters (Fig 1). Populations east of approximately -100° longitude have positive eigenvalues, while those west of -100° longitude have negative eigenvalues for the first axis of the PCA. The second axis separates populations from the Midwest (Minnesota and Wisconsin) from the rest of the eastern populations (Fig 1; yellow circles). The third axis of the PCA separates most of the western US populations (green circles) from Canadian western populations (Fig 2). Populations from New Mexico and Colorado are the most separated from the rest of the western populations, in the top left quadrant. The first, second and third principal components explain a combined 86% of the variability.

**Fig 3:**
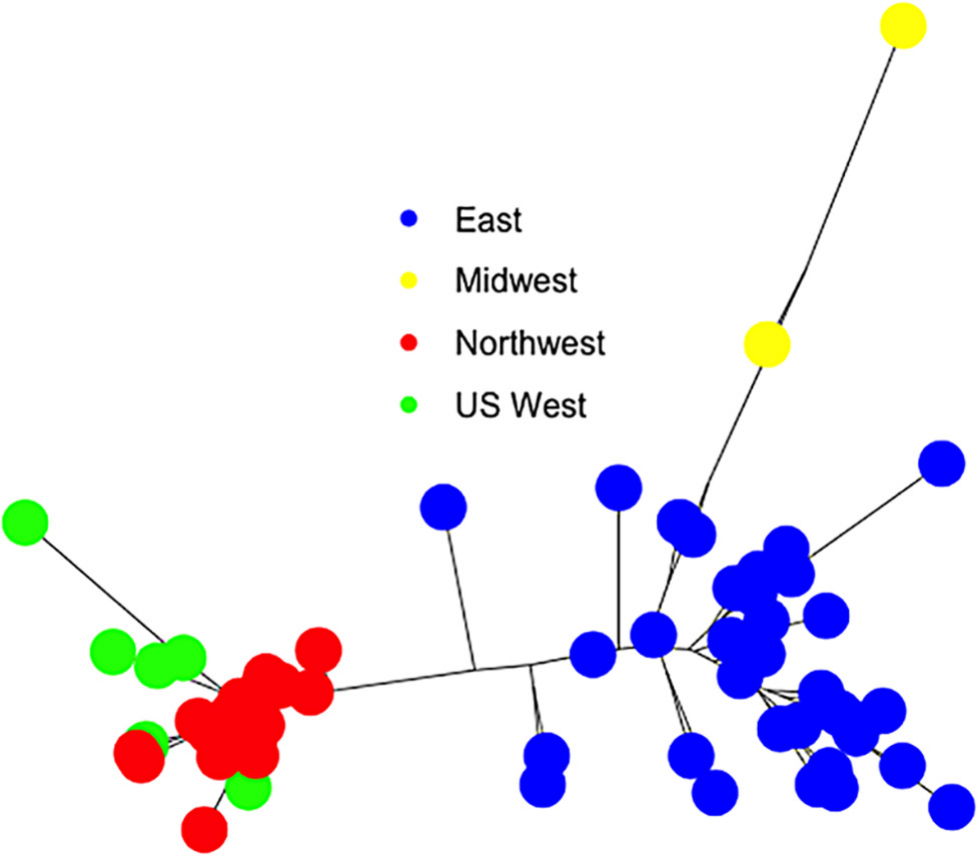
Neighbor-joining tree based on Nei’s unbiased pairwise genetic distances derived from the allele frequency at 31 SNPs among 66 North American populations of *Cronartium ribicola*. East, Midwest, Northwest and US West refer to regions in S1 Table.

The neighbor-joining analysis using Nei’s unbiased genetic distances yields a topology that also groups populations according to their geographic origin (Fig 3). The eastern *C*. *ribicola* populations form an extensive cluster with variable branch lengths. The populations from the Midwest are clearly within the eastern cluster, but are separated by long branches. By contrast, western populations form a cluster with short branches. Only the US western populations display longer branches, notably the population from New Mexico.

**Fig 4:**
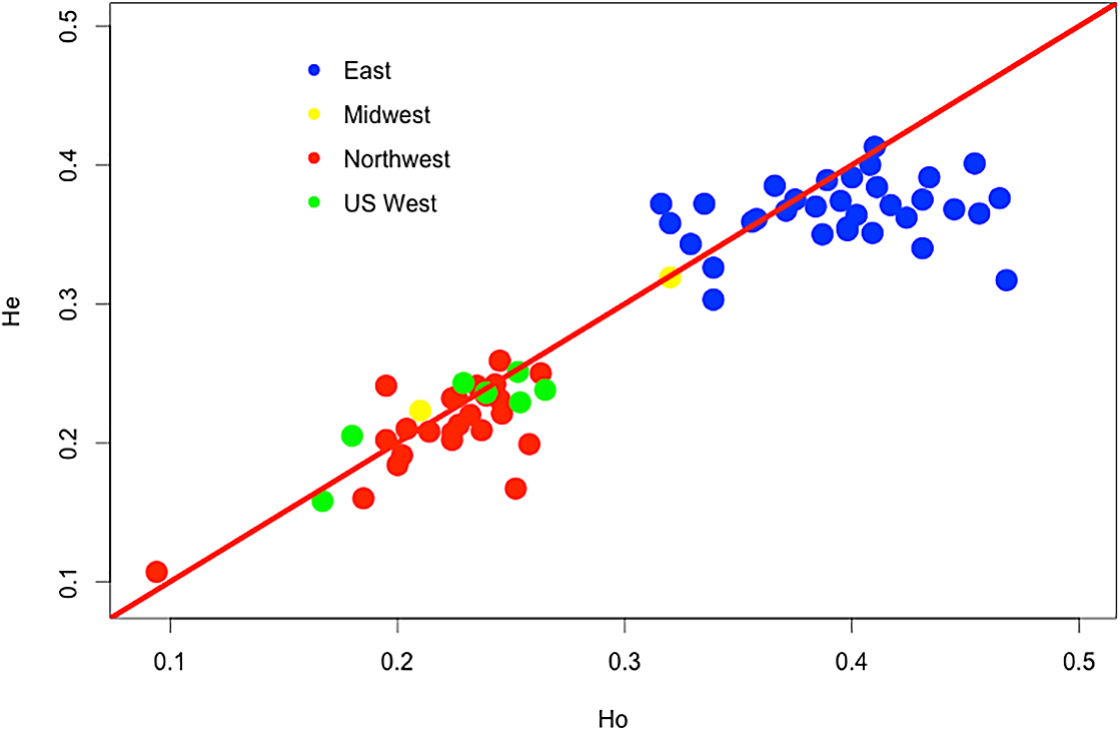
Observed and expected heterozygosity for 66 North American populations of *Cronartium ribicola*. The red line represents identical values for expected and observed heterozygosity. Values above the red line represent populations where inbreeding is observed and those under the line represent populations with excess of observed heterozygotes.

An AMOVA shows a large genetic variance (Φ_ct =_ 0.233, p<0.001) attributed to sampling of eastern and western populations (Table 1). Heterozygosity is consistently higher in eastern populations than in western ones (Fig 4; S3 Table). The lowest heterozygosity within the eastern region is observed in Minnesota and is within the range found in western populations. The lowest heterozygosity overall is observed in New Mexico. The only population with a significant deviation from Hardy-Weinberg equilibrium is Smithers, the northernmost population of *C*. *ribicola* in North America, which displays a deficiency of heterozygotes (S3 Table).

**Table 1:** Analysis of molecular variance for *Cronartium ribicola* populations sampled across geographic regions, hosts and landscapes in North America.

Comparison^a^	Among groups (%)	Among samples within groups (%)	Φ_ct_ ^b^
**Eastern vs western North America**	23.3	5.1	0.233**
**Coastal vs Interior Western North America**	1.0	5.9	0.010**
**Natural stands vs plantations** ^**c**^	1.2	4.4	0.012**
**Host pine species** ^**c**^	0.0	4.2	0.000^ns^
**Elevation** ^**c**^	0.0	4.3	-0.001^ns^

^a^Comparison among and within groups of populations arranged by geographic origin, type of stand, pine host and elevation.

^b^Probability that the observed value is larger than the value calculated following permutation (**p<0.01). Φ_c**t**_ values were tested by permuting whole populations among groups.

^c^See S1 Table for details. Three pine species in western populations were considered: *P*. *monticola*, *P*. *albicaulis*, and *P*. *flexilis* **.** Other pine species (*P*. *strobus*, *P*. *strobiformis*) would have generated confounded effect of host and geography.

To determine population structure without *a priori* assumption of population membership the software *Structure* was used to assign individuals to K clusters. The optimal value of K was estimated to be 2, based on the ΔK/K values. A total of 986 individuals from western North America have a low level of admixture and a high proportion of assignment to the same cluster (red bars in S1 Fig); 306 individuals from eastern North America were assigned to a second cluster (blue bars in S1 Fig), and displayed variable levels of admixture. Increasing K values above two did not reveal additional structure (results not shown).

**Fig 5:**
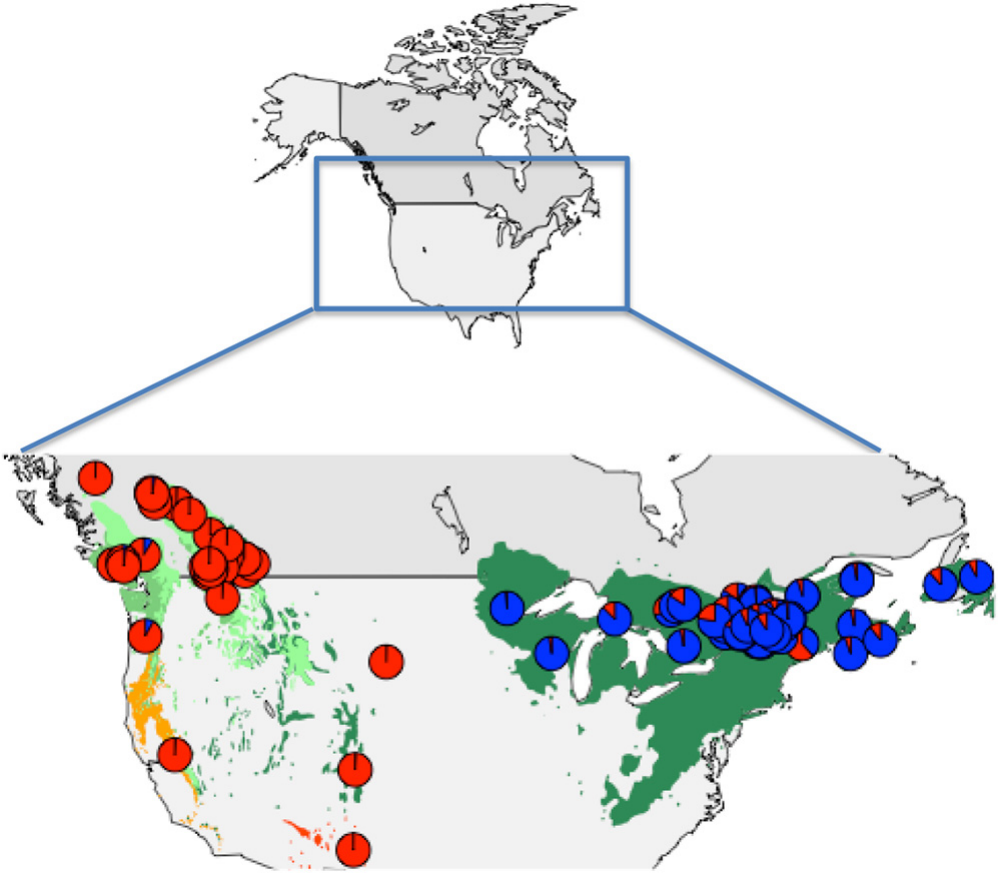
Distribution of white pines, the aecial host of *Cronartium ribicola*, in North America. Each color on the map represents the range of a different white pine species. For each sampled population, the proportion of individuals assigned to each of the two clusters identified in Structure is plotted in a pie chart. The bar represents a barrier to gene flow identified using the Monmonier algorithm.

When the proportion of assignment to the two genetic clusters is averaged for each population and overlaid on a geographic map, there is a clear spatial divide between eastern and western North America. This divide also corresponds to the absence of white pines in central North America (Fig 5). Populations east of the Great Plains sampled on eastern white pines have a large proportion of assignment to the eastern (blue) cluster. Populations west of the Great Plains, but including South Dakota, sampled on all six western white pine species comprised a large proportion of individuals assigned to the western (red) cluster (Fig 5). Individuals from South Dakota, the easternmost population within the western cluster, and from Minnesota, the westernmost population within the eastern cluster, are assigned to the western and eastern clusters, respectively, and little or no admixture was observed (Fig 5).

### Impact of landscape features and host distribution western populations of white pine blister rust

To investigate the spatial distribution of the genetic diversity, an isolation-by-distance analysis was conducted. There is a strong and highly significant correlation between genetic and geographic distances when all populations are analyzed (Mantel test R = 0.73, p<0.001; S2 Fig). However, the plot of genetic vs geographic distances reveals a bimodal distribution that corresponds to assignment to the eastern or western cluster (S2 Fig). When an isolation-by-distance analysis is conducted separately for each cluster (excluding the outlier populations of New Mexico and Minnesota), no significant association is observed between geographic and genetic distance matrices, indicating that isolation-by-distance is not statistically supported within the two genetic clusters (S3 Fig).

To further investigate and discern cryptic spatial patterns that may be associated with landscape features [30], we conducted a spatial Principal Component Analysis (sPCA). The sPCA enables the detection of a global spatial pattern, with the first eigenvalue having a large spatial autocorrelation and a strong genetic variance. When the analysis was conducted separately for eastern and western populations, the analysis revealed a weak spatial signal for eastern populations but a strong signal for western populations (results not shown).

**Fig 6:**
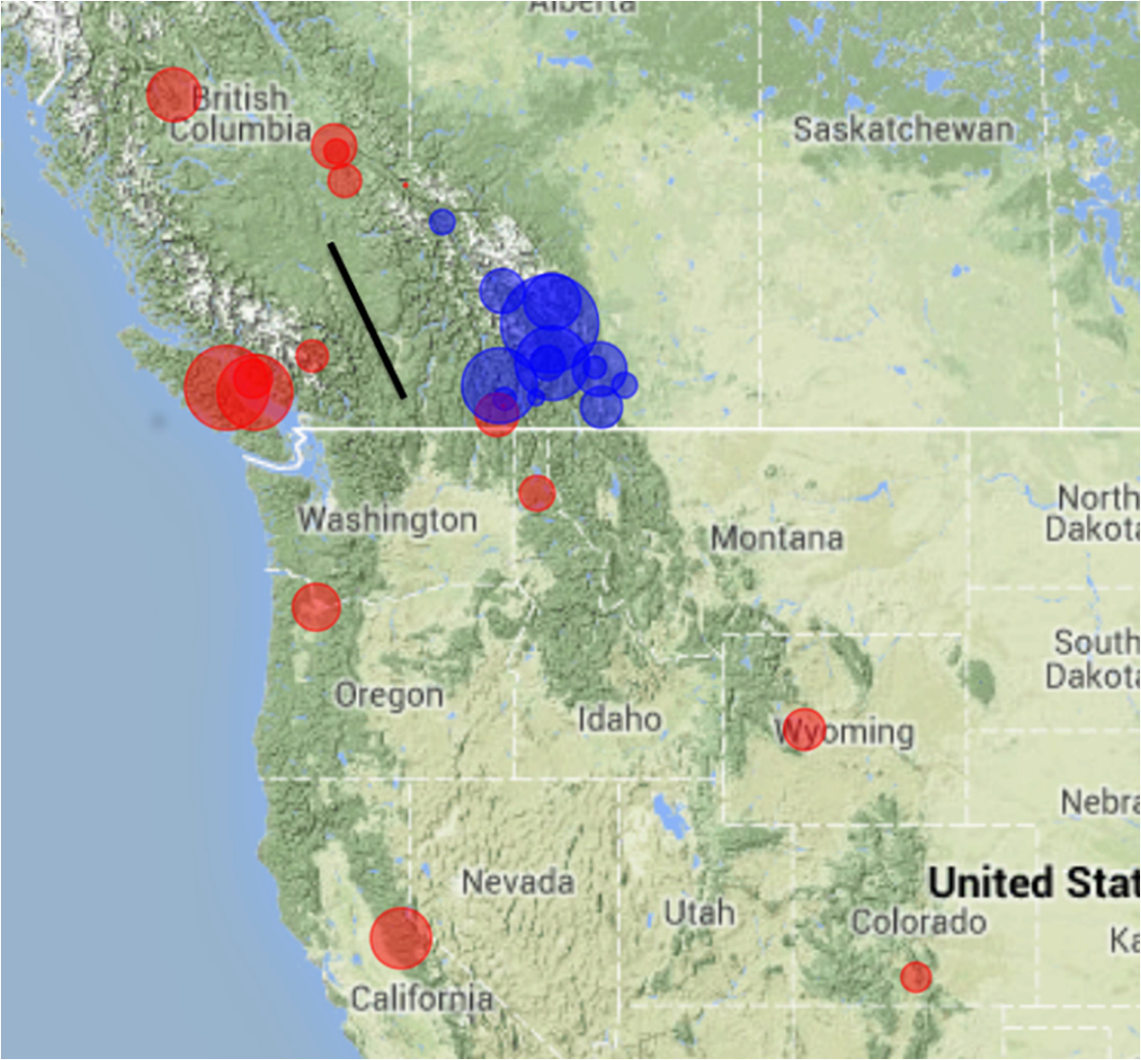
Spatial principal component analysis of western *Cronartium ribicola* populations using Delaunay triangulation as connection network. The first positive eigenvalue component of the sPCA, corresponding to global structure, was plotted on the map. Each population is represented by a blue (positive scores) or red (negative scores) circle of a size proportional to the score loading. The bar represents a barrier to gene flow identified using the Monmonier algorithm in the R package *Adegenet*.

Scores for each western population were plotted on a geographic map (Fig 6). There is a clear demarcation between populations in the Canadian Rocky Mountains (in blue, displaying high positive scores) and the remainder of the western populations, including coastal BC, northern interior BC and western USA (in red, displaying negative scores). This pattern corresponds both to landscape features (the divide between the Rocky Mountains and the Coastal range) and to the absence of pine hosts (Fig 4). This was confirmed by the AMOVA revealing a highly significant genetic differentiation (Φ_ct =_ 0.010, p<0.001) attributed to sampling coastal versus interior populations of *C*. *ribicola* (Table 1).

### Host distribution and landscape features correspond to barriers to migration

To assess the presence of barriers to migration, we used the Monmonier algorithm to detect genetic boundaries among our geo-referenced populations, based on spatial connectivity among locations and genetic distances among the populations. The analysis revealed a path through the population network that clearly separates eastern and western populations and represents a barrier to migration (Fig 5 and S4 Fig). The geographic coordinate through which the barrier runs defines a region in the Canadian Prairies and the US Great Plains where pine hosts are absent (Fig 5).

The Monmonier algorithm was similarly used to assess the presence of a barrier to gene flow among western populations. The presence of a barrier to migration corresponding to the absence of pine hosts and/or the presence of landscape features in the Rocky Mountains was revealed (Fig 5 and S4 Fig).

### Type of stand, but not host or elevation, influence population structure

No significant genetic differentiation was observed between populations sampled at high and low elevations or on different host species (Table 1). However, the AMOVA revealed a highly significant genetic differentiation between *C*. *ribicola* sampled in natural stands and in plantations (Table 1). To further investigate this pattern, we compared assignment to the two genetic clusters identified by the Structure analysis. A significantly higher proportion of individuals were assigned to the eastern than to the western genetic cluster in plantations than in natural stands (Table 2). This pattern was seen in both eastern and western North American *C*. *ribicola* populations.

**Table 2:** Assignment of individuals to two genetic clusters in natural stands and plantations of white pines in eastern and western North America.

Region	Sampling	Assignment (%)^a^	
		Eastern cluster	Western cluster
East	Plantation	92.5	7.5
	Natural stands	85.4	14.6
West	Plantation	3.0	97.0
	Natural stands	0.4	99.6

^a^Cluster assignment is significantly different between plantations and natural stands (t-test p-value<10^–7^).

## Discussion

### The impact of colonization history on white pine blister rust

*Cronartium ribicola* is an invasive alien pathogen in North America. The history of introduction of this pathogen is well documented and provides a useful framework for comparing the impact of colonization history on the population structure of invasive pathogens. Population genetic features at the continental and regional scales reflect contrasting colonization history of the pathogen in eastern and western North America. Our observation of larger genetic diversity in eastern than western *C*. *ribicola* populations supports the scenario of repeated or multiple introduction events that occurred in eastern North America but of limited introduction events in western North America [23].

The first published record of *C*. *ribicola* in North America is on currant in New York State in 1906 [5]. Shortly after, the pathogen was commonly found on pine seedlings in northeastern states [7]. Records show that the pathogen was introduced repeatedly over several years. More than seven million seedlings were imported to over 220 locations in eastern North America from one severely infected nursery in Germany and by 1911, the rust was present in New York, Vermont and Connecticut [8]. By contrast, there is a single documented introduction of the pathogen in western North America, in 1910, from a French nursery to Vancouver. Shortly after, it was found in Washington in 1913, in northern Oregon in 1918 as well as in southern Oregon and California in 1930 [10].

Our results do not support that hypothesis that the rapid spread of the disease observed in western North America is attributed to multiple introduction events [10]. The *C*. *ribicola* populations in BC and western US display similar genetic composition and diversity, and all individuals have a high rate of assignment to the same genetic cluster. Multiple introductions from Europe into western North America should have generated a pattern of genetic diversity similar to that observed in eastern North America; we did not observed such a pattern.

An alternative but not mutually exclusive explanation for the narrow genetic diversity observed in western North America could be that a founder effect had already taken place in France, the only recorded introduction event of *C*. *ribicola* in western North America. The pathogen was first detected in 1856 on *Ribes nigrum* in Estonia, then on eastern white pine in Finland and, subsequently, in Germany. One German nursery was the source of the pathogen in England and other German and European locations [31]. The pathogen is reported to have reached France later, in 1889 [32]. French nurseries had low levels of infection and represented marginal populations on the edge of the pathogen’s geographic distribution, even as late as 1922 and 1929, i.e. after a quarantine prevented seedling importation into North America [31].

### Disjointed populations of white pine blister rust display features of founder effects

Some of the white pines in western North America occur in fragmented patches. This could explain why new colonization events were reported several decades after the initial introduction in BC. The disease was reported for the first time in New Mexico in the 1970s [33], Wyoming in 1978 [34], South Dakota in 1992 [35], North Dakota in 1993 [36] and Colorado in 1998 [37]. Our analyses show that these colonization events likely represent expansions from existing western sources and not from eastern ones. Populations sampled in Colorado and Wyoming, where introductions are recent, have heterozygosity values within the average observed in western populations. By contrast, marginal populations in New Mexico and South Dakota have the lowest heterozygosity in North America. This suggests founder effects following introduction by a few individuals.

The population of *C*. *ribicola* sampled in New Mexico represents the southernmost and most isolated occurrence of the pathogen in North America. It was collected on *Pinus strobiformis*, the southwestern white pine, which has a limited distribution in the mountains of western Texas, New Mexico, Arizona, and southwestern Colorado. It is hypothesized that either the pathogen was introduced into New Mexico from nursery seedlings or that spores were blown from across the Sierra Nevada [12,23]. Both scenarios could have resulted in a population bottleneck. Additional sampling, in particular on the west side of the Sierra Nevada, would allow testing of these different hypotheses.

The *C*. *ribicola* population from Minnesota also displays a signature of reduced genetic diversity. This population constitutes an exception to the pattern of genetic diversity observed in eastern and western North America. Even though it is clearly assigned to the eastern genetic cluster, it displays genetic diversity within the range observed for western populations. We propose two hypotheses to explain these observations: 1) Minnesota represents the westernmost occurrence of eastern white pine and spread of *C*. *ribicola* from other eastern populations is reduced by the prevailing westerly winds; or 2) the Minnesota population originates from a distinct introduction event and did not admix with other eastern or western populations.

There is support for both of these hypotheses. The initial rust outbreak in Minnesota and western Wisconsin was traced to a nursery in the St. Croix valley between Minnesota and Wisconsin that had received a limited number of eastern white pines from the same German nursery that contributed most of the seedlings in eastern North America [8] [38]. These seedlings were inspected for the presence of disease and symptomatic trees were presumably removed; this could have reduced the population size, creating a founder population.

One intriguing fact that could support the second hypothesis is a report of *C*. *ribicola* in Kansas in the late 1800s on currant [5]. This raises the possibility that the pathogen was introduced as an asexual population on *Ribes*, outside of the distribution range of the pine host, resulting in reduced genetic diversity.

### The absence of host connectivity represents a barrier to white pine blister rust migration

Host connectivity likely plays an important role in the dispersal of the pathogen and in shaping the populations. As *C*. *ribicola* is a biotrophic fungus, a host gap constitutes an effective barrier to migration. The eastern and western genetic populations of white pine blister rust defined by our analyses overlap with the distribution ranges of white pines, the aecial hosts of the pathogen. We identified a barrier to gene flow that traverses the Canadian Prairies and extends into the Great Plains, where there are no naturally occurring white pines [23].

Long-distance spore dispersal has been well documented for many fungal species, and in particular for rusts [39,40]. Examples of single long-distance jumps of hundreds of kilometers are rare but have been observed: climate and wind pattern analysis showed that *Melampsora larici-populina* Kleb. and *Melampsora medusae* Thuem., two poplar rusts, were carried by wind over 3000 km, from Australia to New Zealand [41,42]. *Cronartium ribicola* clearly has the capacity to disperse over long distances [43]. The low genetic differentiation among populations within the eastern and western genetic clusters and the absence of an isolation-by-distance pattern within each genetic cluster indicate that there is sufficient migration among interconnected host stands to overcome founder effects and counteract any population-size fluctuations that would favor genetic drift.

But clearly, the absence of host connectivity can prevent or slow long-distance spread. Some of the white pines found in western North America, such as *P*. *strobiformis* and *P*. *flexilis*, have fragmented distributions separated by dry inter-montane rangeland, creating a network of habitat islands subject to island biogeographic processes [44]. In South Dakota, white pine blister rust occurs within the isolated Black Hills, on limber pines (*P*. *flexilis*). This is the easternmost natural extension of limber pine and it is isolated from the nearest limber pine stand by 160 km and from the nearest known population of *C*. *ribicola* by 240 km [35]. It is approximately 800 km west of the nearest eastern white pine in Minnesota. All individuals from South Dakota and Minnesota were assigned to their respective western and eastern genetic clusters, without evidence of admixture. This suggests that dissemination of the rust across this no-host zone is prevented and that the barrier to gene flow is effective. However, since the South Dakota population was only established in the 1990s [35], and our sampling was limited, it is possible that the rust could eventually overcome this barrier.

Since *C*. *ribicola* is a heteroecious pathogen, it requires alternation between aecial and telial hosts in order to complete its life cycle. The basidiospores, the only *C*. *ribicola* spore type that can infect pines, are produced only on the telial hosts, i.e. angiosperms in the families Grossulariaceae and Orobanchaceae [15,17,18,45]. The urediniospores which are also produced on the telial hosts can re-infect telial hosts, creating a repeating cycle of sporulation and infection, while aeciospores produced on the pine host are involved in long range dispersal [43]. It is possible that commercial cultivation of *Ribes nigrum*, the black currant, could increase host connectivity and eventually lead the to the expansion of white pine blister rust across the eastern and western barriers that we identified.

### Western populations of white pine blister rust display cryptic spatial patterns

White pine blister rust is exposed to more landscape features and climatic variations as well as to a broader range of host species in western than in eastern North America. Thus, we conducted intensive sampling in western Canada to cover the breadth of landscapes (coastal, interior, mountains), climates (maritime, continental) and hosts (three white pine species) to investigate whether there are additional drivers of population structure.

We identified a cluster of populations in the Rocky Mountains that is genetically differentiated from the remainder of the western populations. These populations could be undergoing drift maintained by a barrier to gene flow that corresponds roughly to an area where host connectivity is discontinued. This area also has some landscape features, such as the Rocky Mountains and the Trench, which separates the Rocky Mountains on the eastern slopes from the Columbia Mountains and the Cassiar Mountains westward.

The genetic differentiation that we observed between *C*. *ribicola* sampled in natural stands and plantations is indicative that there is some additional dissemination of the pathogen during the regeneration process. The most likely cause of this differentiation is the additional introduction of the pathogen from nurseries. This disease is difficult to detect in seedlings since infections can take up to four years to generate visible symptoms. Since many seedlings are planted after 2 years of growing in the nurseries, it is possible that infected asymptomatic seedlings are planted, generating populations that are genetically distinct.

Host species association and elevation *per se* did not affect the structure of rust populations. This confirms other reports where *C*. *ribicola* isolated from different aecial and telial hosts did not have different genetic profiles or differ in ability to infect telial hosts (when artificially inoculated) [45]. However, host species association is an important epidemiological parameter to consider for a biotrophic pathogen. Specialization on the aecial (conifer) host was found to be a major driver of speciation in poplar rusts [46]. It is possible that the encounter between *C*. *ribicola* and native North American pines is too recent for this specialization to leave a genetic footprint. We only sampled a small number of genes and it is likely that host specialization involves other genes such as effectors undergoing accelerated evolution leading to speciation [47].

### Implications for management of white pine blister rust

Understanding the population structure of *C*. *ribicola* is important for the management of white pine blister rust. Extensive resources are invested in breeding programs to develop and deploy rust-resistant white pines and black currants [48–51]. Our findings describe strongly differentiated eastern and western genetic populations of the pathogen that are separated by a barrier to gene flow. This barrier seems to be effective and is maintained by the absence of host connectivity. Since different races of the pathogen have been observed in western North America on both pines and *Ribes* [52–56] maintaining this barrier and monitoring the migration of the pathogen will be important to prevent migrations of the pathogen and subsequent shifts in pathogen races that could lead to novel adaptations. The presence of some detectable admixture between populations on either side of the continental barrier suggests the potential for the rust to cross that barrier, possibly aided by anthropogenic activities, such as the movement of white pines or Ribes with cryptic *C*. *ribicola* infections. Monitoring populations of *C*. *ribicola* and testing seedlings before outplanting could prevent additional introduction of migrants with novel genetic backgrounds.

## Supporting Information

S1 Fig Bayesian assignment to each of the two genetic clusters for 1,292 individuals of *Cronartium ribicola* genotyped at 31 SNP loci. (TIFF)

S2 Fig Test of isolation-by-distance for 66 populations of *Cronartium ribicola* sampled across North America and genotyped at 31 SNP loci. A Mantel correlation between genetic and geographic distance was generated for each pair of populations and a correlation coefficient was measured (A). A Monte-Carlo test was conducted to obtain 1000 simulations and plot the random distribution of the data (B). The observed value was added.
(TIFF)

S3 Fig Plot of the genetic and geographic distances for 35 eastern (A) and 31 western (B) populations of *Cronartium ribicola* from North America. (TIFF)

S4 Fig Identification of barriers to gene flow among North American (A) and western Canadian (B) populations of *Cronartium ribicola* sampled in this study. (TIFF)

S1 Table Provenance of *Cronartium ribicola* samples. (DOC)

S2 Table Primer and probe sequences for iPlex assays *Cronartium ribicola*. (DOC)

S3 Table Population genetic parameters in populations of *Cronartium ribicola* sampled across geographic, landscape and host range. (DOC)

S4 Table Lag vector of principal component for western populations of *Cronartium ribicola*. Vector values were averaged over populations and used to create the map in Fig 6.
(XLSX)
