# Supplementary figures and images for: Colonization History, Host Distribution, Anthropogenic Influence and Landscape Features Shape Populations of White Pine Blister Rust, an Invasive Alien Tree Pathogen
Source: PLoS One. 2015 May 26;10(5):e0127916. doi: 10.1371/journal.pone.0127916 (PMC4444259; doi:10.1371/journal.pone.0127916)

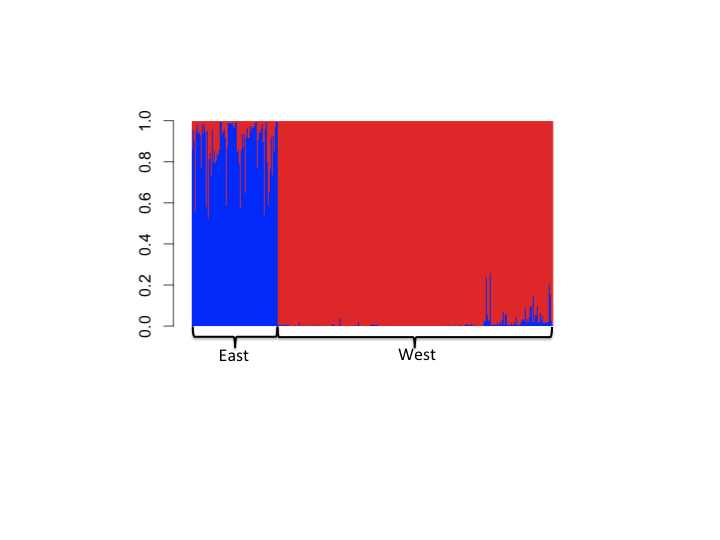

Supplement: S1 Fig — (TIFF) [file pone.0127916.s001.tiff]

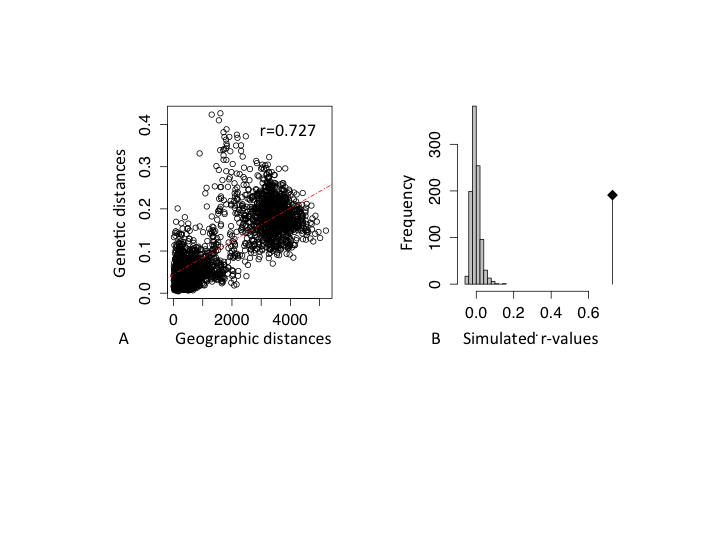

Supplement: S2 Fig — A Mantel correlation between genetic and geographic distance was generated for each pair of populations and a correlation coefficient was measured (A). A Monte-Carlo test was conducted to obtain 1000 simulations and plot the random distribution of the data (B). The observed value was added. (TIFF) [file pone.0127916.s002.tiff]

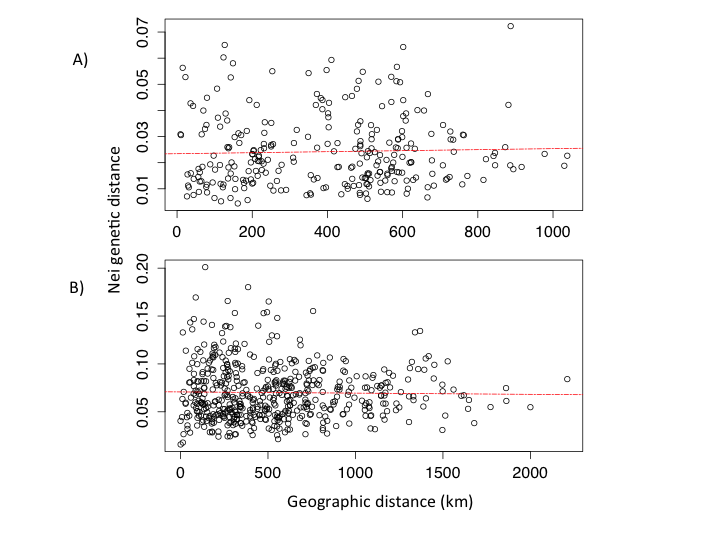

Supplement: S3 Fig — (TIFF) [file pone.0127916.s003.tiff]

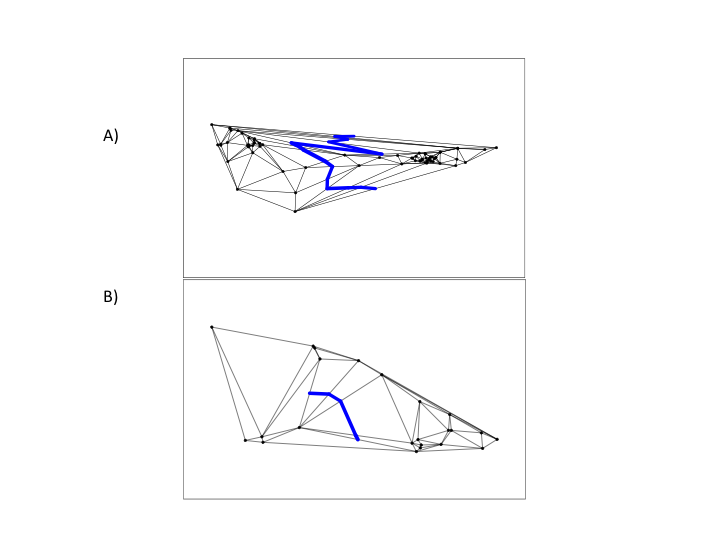

Supplement: S4 Fig — (TIFF) [file pone.0127916.s004.tiff]
