# Supplementary material for: Colonization History, Host Distribution, Anthropogenic Influence and Landscape Features Shape Populations of White Pine Blister Rust, an Invasive Alien Tree Pathogen
Source: PLoS One. 2015 May 26;10(5):e0127916. doi: 10.1371/journal.pone.0127916 (PMC4444259; doi:10.1371/journal.pone.0127916)
Supplement: S1 Table — (DOC) [file pone.0127916.s005.doc]

S1 Table. Provenance of *Cronartium ribicola* samples

| **Population Name** | **State or Province** | **Region** | **Host** | **Stand Type** | **Geographic coordinates** | |
| --- | --- | --- | --- | --- | --- | --- |
|  |  |  |  |  | Longitude | Latitude |
| **Gander River** | Newfoundland | East | *P. strobus* | Natural | -54.813 | 49.026 |
| **Little Grand Lake** | Newfoundland | East | *P. strobus* | Plantation | -57.743 | 48.559 |
| **Perch Lake** | Nova Scotia | East | *P. strobus* | Plantation | -65.101 | 44.495 |
| **Trafalgar** | Nova Scotia | East | *P. strobus* | Plantation | -62.660 | 45.288 |
| **Moncton** | New Brunswick | East | *P. strobus* | Plantation | -64.803 | 46.116 |
| **Quebec** | Quebec | East | *P. strobus* | Natural | -72.608 | 46.449 |
| **Belleterre** | Quebec | East | *P. strobus* | Natural | -78.691 | 47.382 |
| **Bolton Sud** | Quebec | East | *P. strobus* | Natural | -72.369 | 45.160 |
| **Chesterville** | Quebec | East | *P. strobus* | Natural | -71.862 | 45.963 |
| **Corte-Réal** | Quebec | East | *P. strobus* | Natural | -64.600 | 48.908 |
| **Cowansville** | Quebec | East | *P. strobus* | Natural | -72.750 | 45.208 |
| **Durham Sud** | Quebec | East | *P. strobus* | Plantation | -72.340 | 45.660 |
| **La Tuque** | Quebec | East | *P. strobus* | Natural | -72.786 | 47.434 |
| **Lac Brome** | Quebec | East | *P. strobus* | Plantation | -72.517 | 45.217 |
| **Maniwaki** | Quebec | East | *P. strobus* | Natural | -75.967 | 46.375 |
| **Plessisville** | Quebec | East | *P. strobus* | Plantation | -71.772 | 46.220 |
| **Plessisville-97** | Quebec | East | *P. strobus* | Plantation | -71.773 | 46.220 |
| **Quatre-Chemins** | Quebec | East | *P. strobus* | Natural | -70.509 | 46.230 |
| **Rivière Lièvre** | Quebec | East | *P. strobus* | Natural | -75.122 | 46.869 |
| **Ruisseau Tortue** | Quebec | East | *P. strobus* | Natural | -76.185 | 46.417 |
| **Saint-Alexis-de-Montcalm** | Quebec | East | *P. strobus* | Plantation | -73.621 | 45.934 |
| **Saint-Cyprien** | Quebec | East | *P. strobus* | Plantation | -69.019 | 47.896 |
| **Saint-Cyprien97** | Quebec | East | *P. strobus* | Plantation | -69.019 | 47.896 |
| **Sainte-Camille-de-Bellechasse** | Quebec | East | *P. strobus* | Natural | -70.219 | 46.494 |
| **Sainte-Marguerite-de-Bellechasse** | Quebec | East | *P. strobus* | Plantation | -70.219 | 46.494 |
| **Saint-Just-de-Bretenières** | Quebec | East | *P. strobus* | Plantation | -70.084 | 46.574 |
| **Saint-Louis-de-France** | Quebec | East | *P. strobus* | Natural | -72.599 | 46.421 |
| **Sunny Bank** | Quebec | East | *P. strobus* | Plantation | -64.593 | 48.830 |
| **Tingwick** | Quebec | East | *P. strobus* | Natural | -71.944 | 45.881 |
| **Maine** | Maine | East | *P. strobus* | NA | -69.083 | 45.083 |
| **Minden** | Ontario | East | *P. strobus* | Natural | -78.724 | 44.925 |
| **Temagami** | Ontario | East | *P. strobus* | Natural | -79.782 | 47.067 |
| **Sault Ste. Marie** | Ontario | East | *P. strobus* | Natural | -84.350 | 46.533 |
| **Wisconsin** | Wisconsin | Midwest | *P. strobus* | NA | -89.500 | 44.500 |
| **Minnesota** | Minnesota | Midwest | *P. strobus* | Plantation | -93.137 | 47.163 |
| **Banff** | Alberta | Northwest | *P. albicaulis* | Natural | -115.943 | 51.300 |
| **Carbondale River Road** | Alberta | Northwest | *P. albicaulis* | Natural | -114.536 | 49.380 |
| **Plateau Mountain** | Alberta | Northwest | *P. albicaulis* | Natural | -115.945 | 50.191 |
| **Porcupine Hills** | Alberta | Northwest | *P. flexilis* | Natural | -113.880 | 49.787 |
| **Slacker Creek** | BC Interior | Northwest | *P. albicaulis* | Natural | -114.591 | 50.081 |
| **Cranbrook** | BC Interior | Northwest | *P. albicaulis* | Natural | -116.074 | 50.193 |
| **Puddingburn** | BC Interior | Northwest | *P. albicaulis* | Natural | -116.407 | 49.560 |
| **Nelson** | BC Interior | Northwest | *P. albicaulis* | Natural | -117.302 | 49.534 |
| **Red Mountain** | BC Interior | Northwest | *P. albicaulis* | Natural | -117.344 | 49.402 |
| **Quartz Gravel Pit** | BC Interior | Northwest | *P.monticola* | Natural | -117.368 | 51.490 |
| **Bombi Summit** | BC Interior | Northwest | *P.monticola* | Natural | -117.521 | 49.238 |
| **Little Slocan** | BC Interior | Northwest | *P.monticola* | Natural | -117.716 | 49.616 |
| **Kootenays** | BC Interior | Northwest | *P.monticola* | Natural | -116.019 | 50.908 |
| **Springer Creek** | BC Interior | Northwest | *P.monticola* | Natural | -117.450 | 49.779 |
| **McBride** | BC Interior | Northwest | *P. albicaulis* | Natural | -120.130 | 53.337 |
| **Valemount** | BC Interior | Northwest | *P.monticola* | Natural | -119.083 | 52.701 |
| **Prince George1** | BC Interior | Northwest | *P.monticola* | Plantation | -122.174 | 53.992 |
| **Prince George2** | BC Interior | Northwest | *P.monticola* | Plantation | -121.870 | 53.413 |
| **Prince George3** | BC Interior | Northwest | *P.monticola* | Plantation | -122.106 | 53.907 |
| **Smithers** | BC Interior | Northwest | *P. albicaulis* | Natural | -126.747 | 54.850 |
| **Pemberton** | BC Coast | Northwest | *P.monticola* | Plantation | -122.800 | 50.321 |
| **Texada** | BC Coast | Northwest | *P.monticola* | Plantation | -124.438 | 49.654 |
| **Powell River** | BC Coast | Northwest | *P.monticola* | Plantation | -124.488 | 49.903 |
| **Mt. Washington** | BC Coast | Northwest | *P. monticola* | Natural | -125.234 | 49.740 |
| **Idaho** | Idaho | US west | NA | NA | -116.376 | 47.754 |
| **Oregon** | Oregon | US west | *P. lambertiana* | Natural | -122.682 | 45.520 |
| **South Dakota** | South Dakota | US west | *P. flexilis* | Natural | -103.692 | 44.002 |
| **Wyoming** | Wyoming | US west | *P. flexilis* | NA | -108.734 | 43.019 |
| **California** | California | US west | *P. lambertiana* NA | | -120.262 | 38.505 |
| **Mosca Pass** | Colorado | US west | *P. aristata* | Plantation | -105.550 | 37.629 |
| **New Mexico** | New Mexico | US west | *P. strobiformis* | Natural | -105.691 | 32.885 |

NA: information not available
