# Supplementary material for: Colonization History, Host Distribution, Anthropogenic Influence and Landscape Features Shape Populations of White Pine Blister Rust, an Invasive Alien Tree Pathogen
Source: PLoS One. 2015 May 26;10(5):e0127916. doi: 10.1371/journal.pone.0127916 (PMC4444259; doi:10.1371/journal.pone.0127916)
Supplement: S3 Table — (DOC) [file pone.0127916.s007.doc]

| S3 Table. Population genetic parameters in populations of *Cronartium ribicola* sampled across geographic, landscape and host range | | | | |
| --- | --- | --- | --- | --- |
| **Population** | **N** | **Ho** | **He** | **F** |
| NewfoundlandGanderRiver | 7 | 0.329 | 0.370 | 0.111 |
| NewfoundlandLittleGrandLake | 9 | 0.358 | 0.383 | 0.065 |
| NovaScotiaPerchLake | 7 | 0.424 | 0.390 | -0.087 |
| NovaScotiaTrafalgar | 14 | 0.356 | 0.372 | 0.043 |
| NewBrunswick | 12 | 0.387 | 0.366 | -0.057 |
| QC | 9 | 0.398 | 0.377 | -0.056 |
| QCBelleterre | 4 | 0.468 | 0.370 | -0.265 |
| QCBoltonSud | 5 | 0.431 | 0.394 | -0.094 |
| QCChesterville | 8 | 0.375 | 0.400 | 0.063 |
| QCCorte-Real | 8 | 0.431 | 0.400 | -0.077 |
| QCCowansville | 8 | 0.454 | 0.428 | -0.061 |
| QCDurhamSud | 5 | 0.402 | 0.404 | 0.005 |
| QCLaTuque | 10 | 0.456 | 0.384 | -0.188 |
| QCLacBrome | 4 | 0.339 | 0.378 | 0.103 |
| QCManiwaki | 6 | 0.400 | 0.427 | 0.063 |
| QCPlessisville | 17 | 0.411 | 0.397 | -0.035 |
| QCPlessisville-97 | 11 | 0.335 | 0.390 | 0.141 |
| QCQuatre-Chemins | 7 | 0.316 | 0.401 | 0.212 |
| QCRiviereLievre | 7 | 0.320 | 0.385 | 0.169 |
| QCRuisseauTortue | 7 | 0.434 | 0.421 | -0.031 |
| QCSaint-Alexis-de-Montcalm | 13 | 0.410 | 0.430 | 0.047 |
| QCSaint-Cyprien | 20 | 0.408 | 0.411 | 0.007 |
| QCSaint-Cyprien-97 | 9 | 0.366 | 0.408 | 0.103 |
| QCSainte-Camille-de-Bellechasse | 9 | 0.465 | 0.399 | -0.165 |
| QCSainte-Marguerite-de-Lingwick | 6 | 0.389 | 0.427 | 0.089 |
| QCSaint-Just-de-Bretenieres | 4 | 0.371 | 0.419 | 0.115 |
| QCSaint-Louis-de-France | 5 | 0.339 | 0.339 | 0.000 |
| QCSunnyBank | 5 | 0.445 | 0.409 | -0.088 |
| QCTingwick | 4 | 0.395 | 0.427 | 0.075 |
| Maine | 8 | 0.398 | 0.390 | -0.021 |
| OntarioMinden | 9 | 0.409 | 0.372 | -0.099 |
| OntarioTemagami | 15 | 0.384 | 0.383 | -0.003 |
| OntarioSault-Sainte-Marie | 12 | 0.417 | 0.388 | -0.075 |
| Wisconsin | 7 | 0.320 | 0.350 | 0.086 |
| Minnesota | 15 | 0.210 | 0.232 | 0.095 |
| ABBanff | 17 | 0.246 | 0.228 | -0.079 |
| ABCarbondaleRiverRoad | 15 | 0.237 | 0.217 | -0.092 |
| ABPlateauMountain | 50 | 0.235 | 0.244 | 0.037 |
| ABPorcupineHills | 20 | 0.204 | 0.216 | 0.056 |
| ABSlackerCreek | 10 | 0.185 | 0.170 | -0.088 |
| Cranbrook | 5 | 0.252 | 0.186 | -0.355 |
| CranbrookPuddingburn | 26 | 0.195 | 0.206 | 0.053 |
| Nelson | 29 | 0.224 | 0.206 | -0.087 |
| NelsonRedMountain | 6 | 0.258 | 0.218 | -0.183 |
| GoldenQuartzGravelPit | 28 | 0.239 | 0.239 | 0.000 |
| BombiSummit | 19 | 0.202 | 0.197 | -0.025 |
| LittleSlocan | 25 | 0.232 | 0.225 | -0.031 |
| Kootnays | 33 | 0.226 | 0.237 | 0.046 |
| SpringerCreek | 28 | 0.263 | 0.255 | -0.031 |
| McBride | 168 | 0.243 | 0.243 | 0.000 |
| Valemount | 100 | 0.224 | 0.233 | 0.039 |
| PrinceGeorge1 | 12 | 0.224 | 0.217 | -0.032 |
| PrinceGeorge2 | 38 | 0.245 | 0.234 | -0.047 |
| PrinceGeorge3 | 32 | 0.245 | 0.263 | 0.068 |
| Smithers | 37 | 0.195 | 0.245 | **0.204** |
| Pemberton | 29 | 0.253 | 0.256 | 0.012 |
| Texada | 78 | 0.229 | 0.245 | 0.065 |
| PowellRiver | 51 | 0.254 | 0.231 | -0.100 |
| Mt.Washington | 52 | 0.239 | 0.239 | 0.000 |
| Idaho | 12 | 0.200 | 0.196 | -0.020 |
| Oregon | 12 | 0.265 | 0.250 | -0.060 |
| SouthDakota | 4 | 0.167 | 0.186 | 0.102 |
| Wyoming | 5 | 0.227 | 0.240 | 0.054 |
| California | 8 | 0.180 | 0.222 | 0.189 |
| ColoradoMoscaPass | 9 | 0.214 | 0.221 | 0.032 |
| NewMexico | 28 | 0.094 | 0.109 | 0.138 |
| N: sample size; Ho: observed heterozygosity; He: unbiased expected heterozygosity; F_IS_, inbreeding coefficient. Global tests for deficiency and excess of heterozygotes were implemented in Genepop. Values considered significant at p < 0.01 (Bonferroni's adjustment to p<0.0001 to account for multiple tests) are indicated in bold. | | | | |
